# Circulating miRNAs in the first trimester and pregnancy complications: a systematic review

**DOI:** 10.1080/15592294.2022.2152615

**Published:** 2022-12-12

**Authors:** Anita Subramanian, Doria Weiss, Kate Nyhan, Andrew Dewan, Anne Marie Z. Jukic

**Affiliations:** a Epidemiology Branch, National Institute of Environmental Health Sciences, Durham, NC, USA; b New York Medical College School of Medicine, Valhalla, NY, USA; c Harvey Cushing/John Hay Whitney Medical Library, Yale University, New Haven, CT, USA; d Department of Chronic Disease Epidemiology and Center for Perinatal, Pediatric and Environmental Epidemiology, Yale School of Public Health, New Haven, CT, USA

**Keywords:** Placenta, miRNA, pregnancy, preeclampsia, preterm, gestational hypertension, growth restriction

## Abstract

Most pregnancy complications originate with early placentation. MicroRNAs (miRNAs) may play an important role in placentation and function as biomarkers of future pregnancy complications. We summarized from the literature all first trimester circulating miRNAs associated with pregnancy complications of placental origin and further identified the miRNAs which have the most evidence as potential early biomarkers for pregnancy complications. We conducted a systematic review following PRISMA reporting guidelines (PROSPERO CRD42020183421). We identified all first trimester serum or plasma miRNAs associated with a pregnancy complication of placental origin (preeclampsia, intrauterine growth restriction (IUGR), gestational hypertension, preterm delivery) and the number of times those miRNAs were identified, as a measure of replication. Twenty-one studies examined 118 unique miRNAs, and 87 were associated with at least one pregnancy complication; preeclampsia was the most common. Seven miRNAs were significantly associated with a pregnancy complication in at least two studies: miR-125b, miR-518b, miR-628-3p, miR-365a-3p, miR-520h, miR-374a-5p, miR-191-5p. Few miRNAs were associated with more than one pregnancy complication: miR-518b and miR-520h with preeclampsia and gestational hypertension, miR-374a-5p and miR-191-5p with preterm birth and preeclampsia. Our systematic review suggests seven miRNAs as potential biomarkers of pregnancy complications. These complications are thought to originate with early placental defects and these miRNAs may also be biomarkers of placental pathology. First-trimester biomarkers of pregnancy complications can facilitate early detection and interventions.

## Introduction

MicroRNAs (miRNAs) are small non-coding RNAs, made up of 18–22 nucleotides that regulate the stability and translation of mRNAs [1]. A single miRNA can regulate the expression of many genes and control the regulation of different processes including cell growth, differentiation, and stress response [2]. MicroRNAs play a part in regulating placental development through trophoblast cell proliferation, apoptosis, migration, invasion, and angiogenesis [3,4]. Previous in vitro studies have shown that miRNA expression in the human placenta, especially in trophoblast cells, is modulated by hypoxia, cell signalling pathways, and epigenetic modifications through promoter methylation [3–5].

Abnormal expression of miRNAs have been found in placentas from preeclamptic pregnancies [6,7]. However, since most pregnancy complications are thought to originate with early placental development [8–10], it is important to assess early changes in miRNA expression to identify predictors. Defects in first trimester placental development can result in pregnancy complications such as preeclampsia [11]. Although studies have examined miRNAs in the serum or plasma, most previous studies have measured miRNAs in the third trimester maternal blood specimens [6,7,12] which limit early detection and prevention of pregnancy complications. Studies that have measured miRNAs in late pregnancy or in the placenta after delivery, have measured miRNAs after the onset of preeclampsia pathology. Assessing miRNAs at these time points may be reflective of the disease response rather than identifying miRNAs that can predict disease development. Conversely, identification of first trimester miRNAs associated with pregnancy complications could improve our understanding of the biological pathways which are associated with complications, and lead to improved clinical detection and therapeutic interventions through their use as biomarkers.

Few studies have discussed the role of miRNAs measured in the serum or plasma during the first trimester and their involvement in the pathogenesis of preeclampsia and other pregnancy complications. To the best of our knowledge, a systematic review summarizing first trimester circulating miRNAs with pregnancy complications has not been conducted. The objectives of this systematic review were to summarize from the literature all first trimester circulating miRNAs associated with pregnancy complications of placental origin and to identify the miRNAs, which have the most evidence as potential early biomarkers for pregnancy complications. The findings from this study can be used to better understand early placental pathology and to target future studies towards miRNAs that are most likely to be useful as predictive biomarkers of pregnancy complications.

## Material and methods

This review followed PRISMA reporting guidelines [13] and was registered in PROSPERO under protocol number CRD42020183421.

### Literature search strategy

The search strategy was designed by a medical librarian (KN) to identify potentially relevant

studies in MEDLINE (on the Ovid platform, including In-Process and PubMed-not-MEDLINE

records), Embase (on the Ovid platform), and Scopus. The search strategy used text words and controlled vocabulary to identify papers, which addressed placental birth outcomes and miRNAs (Table S1). The complete MEDLINE search strategy was peer reviewed by an independent medical librarian. We only included studies published in English, no date limit was imposed, and no study design filters were assigned. Our review included only studies of human data; in databases with subject indexing, citations were reviewed unless they had an animal check tag without a human check tag. Citations from the databases were exported to EndNote and de-duplicated before uploading to Covidence, an online platform for screening and data extraction. EndNote was used for managing records and full texts of articles. Literature searches were conducted in March 2019 and updated in June 2020 and January 2022.

### Eligibility criteria

The review included human observational studies: case–control (nested and non-nested) and cohort studies (prospective and retrospective). Included studies had reported the serum or plasma expression levels of miRNAs (mean/standard deviation (SD) or median/interquartile range (IQR)) in a control group consisting of healthy pregnant women, and in a group with a disorder of placental origin [8–10]: preeclampsia, gestational hypertension, preterm birth, or intrauterine growth restriction (IUGR). We included preterm birth as a complication of placental origin because some cases of preterm birth can occur due to suboptimal decidualization in the first trimester, and sometimes the uterine spiral arteries do not get remodelled thereby affecting blood flow to the foetus [14]. Problems in placental development for implantation such as placenta previa and accreta can also increase the risk of preterm birth [15]. While studies used different terms for growth restriction (IUGR, foetal growth restriction, small-for-gestational age), we have used IUGR in this paper to refer to any type of growth restriction. Studies were excluded if they were reviews without primary data, if the primary outcome was not a placental disorder of pregnancy, or if serum or plasma miRNAs were not measured in the first trimester of pregnancy (≤14 weeks). If data to determine eligibility were missing, such as gestational age at the time of blood draw, the study’s authors were contacted and if we received no response, then the study was excluded from this review (N = 7).

### Dual abstract review

We did two rounds of screening to identify all studies which met the inclusion criteria. In the title-abstract screening stage, each study was screened independently by two authors (DW, AS, AMJ). The decisions were compared, and disagreements were resolved through discussion. Studies considered potentially relevant after title abstract screening moved on to the full-text screening stage. If it was unclear whether a study met the inclusion criteria after completing the title-abstract screening, then these studies were also included to the full-text screening stage.

### Full text review

One author conducted the full-text screening (AS). If any studies were missing information, then the corresponding authors were contacted to obtain further details. Details on whether a study was included or excluded was updated in Covidence. When studies were excluded, the reason for exclusion were noted in Covidence. If eligibility was unclear, discussions were conducted with other authors to resolve issues.

### Data collection

We used a standardized data extraction method to ensure consistency. Data were extracted on author, year of publication, study design, sample size, demographic characteristics of participants such as race/ethnicity, education, and health status/medical history. We extracted details about severity of pregnancy complication (e.g., mild or severe), gestational age at the time of blood draw (in weeks), methods used for evaluation of miRNA expression (e.g., microarray vs. real-time PCR), whether a targeted or a genome-wide analysis was performed, and the mean (SD), median (IQR), or fold change in miRNA expression among those with and without pregnancy complications. Information was abstracted for every miRNA reported in each study even if its association with pregnancy complications was not statistically significant. Then, for every miRNA, we also quantified the number of studies that reported on that miRNA and with which pregnancy complications it was associated. To identify the miRNAs which had the most evidence as potential early biomarkers, we determined the miRNAs which were replicated across studies. As a measure of replication, we identified miRNA that were statistically significantly associated with any pregnancy complication in at least two studies, this could be either the same complication more than once, or two different complications. We present these miRNA metrics both overall and stratified by pregnancy complication. We contacted authors to request a list of all the miRNAs which were identified in studies performing an untargeted analysis but not reported in the findings to help us assess the miRNAs which were not significantly associated with pregnancy complications.

### Risk of bias assessment

The risk of bias was assessed for studies that included replicated miRNAs. We used six criteria for assessing the risk of bias: 1) different gestational ages of serum draw, 2) differences in definitions of pregnancy complication (type of misclassification bias), 3) library preparation bias, 4) base level read count bias, 5) gene length bias (type of detection bias), 6) multiple comparisons/repeating testing bias. The risk of bias for each criterion was rated as low, high, or unknown. The risk of bias was rated as unknown when data for assessment was missing or not presented in the study.

## Results

### Selection of studies

**Figure 1.**
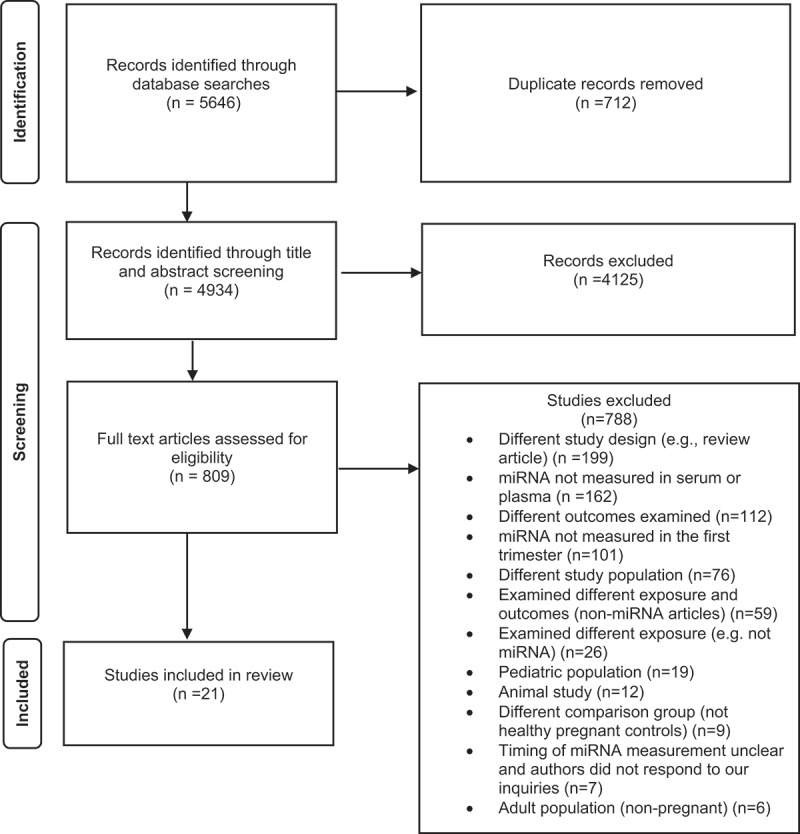
PRISMA flow diagram for studies included in the review.

We identified 5646 studies through database searches, and after removal of duplicates, 4934 studies went through title and abstract screening (Figure 1). Of these, 4125 studies were excluded at the title-abstract screening stage, and 809 studies were eligible for a full-text review. After excluding 788 full-text articles, 21 studies met the eligibility criteria for inclusion in the review. The most common reason for exclusion at the full-text review stage was not measuring miRNA in the first trimester maternal circulating serum or plasma (most measured miRNA in the placenta, trophoblast cells or in the third trimester). The other reasons for exclusion were related to study design such as a review article, not examining a pregnancy outcome of placental origin such as gestational diabetes, ectopic pregnancy, adiposity, folate status, myasthenia gravis, viral infection, foetal macrosomia, sickle cell disease, neural tube defects, congenital heart defects among others. We excluded studies which had a different population such as adults (non-pregnant), paediatric, animal studies, and the ones which did not include a control group of healthy pregnant women or included only a non-pregnant control group.

### Study characteristics

Studies were conducted among a broad range of population groups. Out of the 21 included studies, two were based in the United Kingdom [16,17], three in the Czech Republic [18–20], eight in China [21–28], one in Spain [29], three in Mexico [30–32], one in Russia [33], two in Italy [34,35], and one in Greece [Mavre^36^] (Table S2). Eleven studies conducted a targeted analysis using PCR to measure specific miRNAs [18–21,23–27,30,34], and 10 studies did an untargeted or a genome-wide analysis using a microarray or other methods to measure a large number of miRNAs [16, 17, 22, 28, 29, 32, 33, 35, 31, 36]. The gestational age at the time of the blood draw ranged from 10 to 14 weeks (Table S2). The sample size varied across studies: among the cases (pregnancy complications present) it ranged from 3 to 74, and for controls (healthy pregnancy) it was between 3 and 875.

### Study outcomes

Preeclampsia was examined in 16 studies [18, 20–23, 28–30, 32, 33, 35, 26, 27, 31, 34, 36], and two studies included both preeclampsia and IUGR [18,20]. Preterm birth was examined in one study [16], gestational hypertension in one study [19], and IUGR in five studies [17,18,20,24,25].

### Overall description of identified miRNAs

Across all studies, 118 unique miRNAs were examined, of these 87 miRNAs were associated with at least one pregnancy complication (Table S2). The most frequently studied miRNAs were miR-518b (five studies), miR-125b (four studies), miR-520h (three studies), miR-520a (four studies). Out of the 87 miRNAs which were reported to have associations with pregnancy complications, seven miRNAs were found to be statistically significantly associated with a pregnancy complication in at least two different studies (Table 1). We have presented findings in two ways: details of the miRNAs which have been replicated across studies (Table 1), and a summary of all the miRNAs identified (Table S2).

**Table 1.** MiRNA replication summary: miRNAs that were associated with pregnancy complications in at least two studies.

miRNAs	Total number of studies reporting an association	Outcome (N of studies)	Direction of association^1^	References
miR-125b	3	Preeclampsia (3)	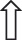	[22,26,34]
miR-518b	3	Preeclampsia (2) Gestational Hypertension (1)	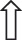 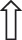	[19,20,35]
miR-628-3p	2	Preeclampsia (2)	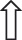	[30,31]
hsa-miR-365a-3p^2^	2	Preeclampsia (2)	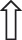 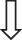	[22,31]
miR-520h	2	Preeclampsia (1) Gestational Hypertension (1)	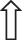 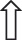	[19,20]
miR-374a-5p	3	Preeclampsia (1) Preterm Birth (1) IUGR (1)	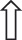 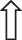 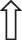	[16,17,22]
miR-191-5p	3	Preeclampsia (1) Preterm Birth (1) IUGR (1)	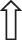 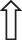 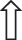	[16,17,22]

IUGR, Intrauterine Growth Restriction.

^1^Arrow represents the direction of association for each outcome.

^2^miRNA expression was reported to increase in one study [31] and decrease in the other study [22].

The miRNAs with the most replications (three studies) were, miR-125b [22,26,34], miR-518b [19,20,35], miR-374a-5p [16,17,22], and miR-191-5p [16,17,22] (Table 1). Among the three studies which replicated miR-125b, two used a targeted PCR [26,34] and one used a microarray [22]. Similarly, two studies used a targeted PCR to measure miR-518b [19,20], and one study used a microarray [35]. All three studies which replicated miR-374a-5p and miR-191-5p were untargeted [16,17,22] and two studies used a Nanostring nCounter assay (measures about 800 miRNA targets and uses hybridization-based methods) for miRNA measurement [16,17] and one used a microarray [22]. The miRNAs that replicated in two studies were miR-520h (both based on PCR) [19,20], miR-628-3p (based on a PCR [30] and a TaqMan low-density array [31]), and hsa-miR-365a-3p (one using microarray [22] and one using a TaqMan low-density array [31]).

### Replication of miRNAs stratified by pregnancy complication

#### Preeclampsia

Of the seven miRNAs that were associated with pregnancy complications in at least two studies, four were associated with preeclampsia in several studies and three were associated with preeclampsia in one study but with other pregnancy complications in at least one other study (Table 1). Among the studies examining miRNAs and preeclampsia, four were targeted and three performed an untargeted analysis. The four miRNAs that replicated across several studies of preeclampsia were miR-125b (three studies), miR518b (two studies), miR-628-3p (two studies), and hsa-miR-365a-3p (two studies) (Table 1).

For miR-125b, first-trimester expression levels were 3- to 4-fold higher between preeclampsia cases and controls [22,26]. Another study reported a significantly higher log miR-125b among preeclamptic compared to healthy pregnant women (−7.16 vs. −8.14, respectively; log concentrations presented) [34]. For miR-518b, first-trimester expression levels were 3- to 5-fold higher in preeclampsia compared to controls [20,35]. For miR-628-3p, both studies reported a 12-fold change in expression between women with mild preeclampsia and controls. Although both studies reporting on miR-628-3p were from the same cohort, one study performed a targeted analysis [32], whereas the other was untargeted [31]. Since we were identifying miRNAs which were replicated across publications, we reported findings on miR-628-3p, though it was from the same cohort. The two studies of hsa-miR-365a-3p reported that expression increased by 0.5 and greater than 2-fold [22,31].

The remaining three miRNAs, miR-520h, miR-374a-5p, and miR-191-5p, replicated across studies of both preeclampsia and other pregnancy complications (Table 1). Expressions of all three miRNAs were higher in the first trimester among women who went on to develop preeclampsia. The mean levels reported for miR-520h indicate a 4-fold increase with preeclampsia compared to controls [20]. A 3-fold change in expression was reported for expression of miR-374a-5p and miR-191-5p between preeclampsia and controls [22].

The other miRNAs that were associated with preeclampsia in one study each were: miR-134, miR-196b, miR-376c, miR-486-3p, miR-590-5p, miR-517-5p, miR-520g, miR-127, miR-423-5p, miR-1233, and miR-144 (Table S2). One study identified twenty nine miRNAs associated with preeclampsia [28], of which, three were higher among preeclamptic women and these miRNAs included hsa‑miR‑1304‑5p, hsa‑miR‑320a, and hsa‑miR‑5002‑5p. The other 26 miRNAs reported in the same study were lower in preeclampsia (Table S2) [28]. Four studies measured miR-520a, and only one study reported an association with preeclampsia (Table S2). Two miRNAs were measured in two studies, but only associated with preeclampsia in one of the studies: miR-210 and miR-126 (Table S2).

### Gestational hypertension

Only one prospective cohort study examined first trimester miRNAs in the maternal circulation with gestational hypertension [19]. In this targeted study, four miRNAs were found to be associated with gestational hypertension, but only two miRNAs were replicated across studies (Table S2). Both miRNAs were associated with preeclampsia in either one (miR-520h) or two (miR-518b) other studies (Table 1). Gestational hypertension was associated with a 13-fold increase in miR-520h and a 4-fold increase in miR-518b.

### Preterm birth

One case–control study performed an untargeted analysis and found nine miRNAs associated with the risk of preterm birth: hsa-miR-150-5p, hsa-miR-374a-5p, hsa-miR-19b-3p, hsa-miR-185-5p, hsa-miR-15b-5p, hsa-miR-191-5p, hsa-miR-93-5p, hsa-let-7a-5p, and hsa-miR-23a-3p [16] (Table S2). Of these nine miRNAs, two miRNAs replicated findings across studies: miR-374a-5p and miR-191-5p, both of which were associated with preeclampsia and IUGR in two other studies (Table 1). Preterm birth was associated with a 6-fold change in first trimester expression of miR-374a-5p and a 5-fold change for miR-191-5p.

### IUGR

Five studies examined IUGR [17,18,20,24,25], and one of these studies looked at IUGR in conjunction with preeclampsia [18]. Across these five studies, eight miRNAs were associated with IUGR. Of these, two miRNAs replicated findings for hsa-miR-374a-5p and miR-191-5p, and both miRNAs were associated with preeclampsia and preterm birth in two other studies (Table 1). First-trimester expression of both the replicated miRNAs was higher in growth restricted infants [17]. Median levels reported for hsa-miR-374a-5p suggest about a 1-fold increase, and ~0.5-fold increase in the expression of miR-191-5p between IUGR infants compared to controls [17]. IUGR was associated with other miRNAs which were not replicated including: let-7d-5p, miR-107, miR-30e-5p, miR-4454+ miR-7975, miR-206 (Table S2).

### Risk of bias in individual studies

The risk of bias for the ten studies that replicated miRNAs are shown in Figure S1. Overall, most studies showed a low risk of bias. Only one study showed a high risk for the multiple comparison bias [26], whereas six studies (Figure S1) had an unclear risk of bias for some of the criteria. One study had an unclear risk for four criteria [35] due to missing or not providing adequate information. One of the reasons for an unclear risk of bias was the gestational age at the time of blood draw. These studies included a range for gestational age such as 12–14 weeks, thereby including both first and early second-trimester measurements. Other reasons for an unclear risk were not providing a definition for the outcome (preeclampsia), inadequate information on methods used to amplify miRNA signal or accounting for gene length bias, and whether the study corrected for multiple comparisons using Bonferroni correction (multiple comparison might have been done but Bonferroni correction was not mentioned).

## Discussion

This systematic literature review identified 21 studies that measured circulating miRNAs in the first trimester and assessed associations of these miRNAs with pregnancy complications of placental origin. First-trimester measurements of miRNAs were associated with pregnancy complications, which occur due to defects in early placentation. Across all studies, 118 unique miRNAs were identified, of which seven were associated with a pregnancy complication in at least two studies. These miRNAs were associated with preeclampsia, gestational hypertension, IUGR, and preterm birth. The miRNAs that were most frequently associated with preeclampsia were miR-125b and miR-518b, followed by miR-628-3p and hsa-miR-365a-3p. Three other miRNAs were associated with both preeclampsia and at least one other pregnancy complication including miR-520h, miR-374a-5p, and miR-191-5p. These seven miRNAs deserve further investigation as potential biomarkers for predicting preeclampsia. Despite their proposed early placental origins, pregnancy complications other than preeclampsia have not been well explored in terms of miRNA expression and prediction. These other complications: growth restriction, preterm birth, and gestational hypertension, should be further studied in relation to first trimester circulating miRNA.

Several miRNAs examined by studies included in this review had higher levels in the first trimester among women who eventually developed preeclampsia compared to healthy controls. We observed that higher miR-125b was associated with preeclampsia in three studies. Higher levels of miR-125b could impact trophoblast invasion and impair endothelial cell function [22,26]. Further, miR-125b can target surface markers involved in cell adhesion, proliferation, and growth [34]. Taken together, this literature suggests that miR-125b may contribute to early placental pathology that leads to preeclampsia. Importantly, expression of this miRNA is detectable in the pregnant person’s plasma or serum and could provide an early biomarker of preeclampsia.

We found studies reporting an increase of miR-518b and miR-520h with preeclampsia and gestational hypertension. These miRNAs belong to the chromosome 19 miR cluster (C19MC), the largest such cluster, and are highly expressed in the trophoblast cells of the placenta [12]. Overexpression of miR-518b can affect trophoblast migration and angiogenesis [6]. Since these miRNAs have a role in placental development, dysregulation of these miRNAs can lead to development of preeclampsia [12]. There is limited research to explain the mechanism contributing to the association between miR-518b and hypertensive disorders of pregnancy. Hromadnikova et al. propose in their study that the mechanisms impacting placentation due to higher levels of miR-518b might be similar to the ones occurring in cancer development and tumour progression [20]. Early placental development and angiogenesis take place primarily in a hypoxic environment, and there might be similarities with tumour pathophysiology [20]. We suggest that miR-518b and miR-520h might have utility as first-trimester biomarkers of hypertensive disorders of pregnancy and future work should focus on measuring miRNAs belonging to C19MC.

We found two studies which reported higher first trimester levels of miR-628-3p in those who eventually developed preeclampsia. Both studies were from the same cohort and examined the same outcome, but one was untargeted, and the other was targeted towards two particular miRNAs. The role of miR-628-3p in pregnancy has not been widely investigated. Martinez-Fierro et al. report in their study that higher levels of miR-628-3p in the first trimester might affect trophoblast functioning [30]. It is known that miR-628-3p targets the gene involved in the regulation of cell processes through its involvement in cell migration and apoptosis [37]. Similarly, higher levels of miR-628-3p in the first trimester might impact the regulation of the gene targeted by miR-628-3p, thereby inhibiting trophoblast functioning and increasing the risk of preeclampsia [30].

Increased expression of hsa-miR-365a-3p was associated with preeclampsia in one study and reduced expression was associated in another study [22,31]. miR-365a-3p has been described as a tumour suppressor in gastric cancer [38], lung cancer [39], pancreatic cancer [40], and other cancers [41]. In patients undergoing in vitro fertilization, miR-365a-3p expression was associated with fertility rate [42], although the association was weakened when adjusted for other miRNAs.

We found that miR-374a-5p and miR-191-5p were associated with preterm birth [16] growth restriction [17], and preeclampsia [22]. All three studies performed an untargeted analysis, but two studies validated expression of miR-374a-5p [16,17], and one study validated miR-191-5p [16] in an independent cohort. However, further investigation of these miRNAs in other populations using a targeted approach would be a logical next step. It is possible that miR-374a-5p is associated with pregnancy complications through its role in downregulation of pro-inflammatory markers [43]. This might also explain its association with multiple pregnancy complications that may share aetiological risk factors. miR-191-5p has been associated with embryo quality, and a higher concentration has been observed in in vitro fertilization (IVF) embryos with aneuploidy, and in the embryos that led to failed IVF cycles [44]. Target prediction software has indicated that miR-191-5p may be involved in cell signalling or apoptosis [44], but the exact mechanisms related to pregnancy or placentation are unknown.

It is well known that early placental development occurs in a hypoxic environment which helps with trophoblast proliferation and formation of the cytotrophoblast layer [45]. It has been suggested that levels of miR-210 increase by about 13-fold in hypoxia-induced cell lines [12]. Anton et al. reported a higher expression of miR-210 in preeclamptic placentas and their findings show that trophoblast invasion is inhibited by miR-210 through protein kinase signalling pathways [46]. Given this in vitro literature, we expected to find several studies that examined miR-210 in the first trimester; however, we only found two studies, and only one study reported a higher level at 12–14 weeks among preeclamptic women [35] (Table S2). Since miR-210 has a potential role in the pathogenesis of preeclampsia and the literature is limited, future investigation of first-trimester miR-210 and pregnancy complications is needed.

Another miRNA of interest which might be associated with preeclampsia but only investigated in two studies in our review, was miR-126. miR-126 may modulate endothelial homoeostasis or increase proliferation, differentiation, and migration of endothelial progenitor cells [6,47]. In addition, miR-126 is pro-angiogenic, and increases vascular endothelial growth factor and fibroblast growth factors through a kinase pathway [12]. We found only one study reporting lower levels of miR-126 in the serum of women with preeclampsia [35] (Table S2). Thus, further research of miR-126 may also be warranted.

The main strength of this review is the comprehensive search of the literature. We did not restrict our search to a specific miRNA and summarized all reported miRNAs. We also summarized the miRNAs which were replicated across studies to identify the miRNAs which had the most evidence as a potential candidate for an early biomarker. Our review had several limitations. First, we were unable to quantify the total number of studies that measured a given miRNA because most studies used an untargeted method of analysis and only reported the miRNAs which were significantly associated with pregnancy complications and not the ones which were null. Thus, if a miRNA was not reported in a given publication, we could not discern if it was not measured at all, or if it was measured, but not significantly associated with the pregnancy complication under study. Second, most of the reviewed studies were small and some studies had a larger sample only in the control group which leads us to interpret the findings with caution. Third, most of the studies were conducted in European populations, and generalizability to other populations may be limited.

In conclusion, this summary of the literature suggests seven miRNAs as potential first-trimester biomarkers for the prediction of placental complications of pregnancy: miR-125b, miR-518b, miR-628-3p, miR-365a-3p, miR-520h, miR-374a-5p, miR-191-5p. Future studies should validate the diagnostic value of these miRNAs by including a larger sample size and diverse populations. Future research could target these miRNAs in the first trimester to determine their predictive ability for preeclampsia, gestational hypertension, preterm birth or IUGR. In addition, understanding the role of these miRNAs in disease pathogenesis should be further examined. Future work should examine the potential use of drugs for the treatment of preeclampsia by targeting these miRNAs. Differences might exist between measuring miRNAs in the serum or plasma and future studies could consider examining whether the replicating matrix leads to reproducibility of findings. Identifying miRNAs which can act as potential non-invasive biomarkers to predict pregnancy outcomes in the first trimester especially among high-risk women can have research implications by identifying signalling pathways for further investigation, and clinical implications by facilitating early detection and timely interventions.

## Supplementary Material

Supplemental Material

## Data Availability

No datasets were generated or analysed during the current study.
