# Supplementary material for: Circulating miRNAs in the first trimester and pregnancy complications: a systematic review
Source: Epigenetics. 2022 Dec 12;18(1):2152615. doi: 10.1080/15592294.2022.2152615 (PMC9980650; doi:10.1080/15592294.2022.2152615)
Supplement: Supplemental Material [file KEPI_A_2152615_SM2135.zip › supplement/Supplementary Information_Revised.docx]

**Table S1: Search Strategy**

**Search histories: March 19, 2019 searches. The same searches were rerun on June 30, 2020.**

**Ovid MEDLINE(R) ALL <1946 to February 20, 2019>**

| 1 | [jukic microRNA search draft 03 MEDLINE] | 0 |
| --- | --- | --- |
| 2 | [microRNA concept -- if we look at specific ones we'll predetermine the findings, so we're casting a broad net here instead of focusing on 210, 223, 126 etc] | 0 |
| 3 | exp microRNAs/ | 63541 |
| 4 | (microRNA* or micro-RNA* or miRNA* or mi-RNA* or miR*).ti,ab,kf. | 161956 |
| 5 | (stRNA* or small temporal rna*).ti,ab,kf. | 31 |
| 6 | or/3-5 | 164985 |
| 7 | [the previous line is the summation of the microRNA concept] | 0 |
| 8 | [and the pregnancy complications of placental origin] | 0 |
| 9 | Pre-Eclampsia/ | 28914 |
| 10 | exp hypertension, pregnancy-induced/ | 34296 |
| 11 | exp pregnancy complications/ | 405148 |
| 12 | Premature Birth/ | 11802 |
| 13 | Fetal Growth Retardation/ | 15411 |
| 14 | exp Infant, Low Birth Weight/ | 32149 |
| 15 | exp Placenta/ | 63584 |
| 16 | (preeclamp* or pre-eclamp*).ti,ab,kf. | 29244 |
| 17 | ((hypertensi* or blood pressure) adj3 (pregnan* or gestation*)).ti,ab,kf. | 13849 |
| 18 | (fetal growth adj (restrict* or retard*)).ti,ab,kf. | 5123 |
| 19 | fgr.ti,ab,kf. | 1570 |
| 20 | (small adj3 gestational age).ti,ab,kf. | 9549 |
| 21 | sga.ti,ab,kf. | 7341 |
| 22 | (low* birthweight* or low* birth weight*).ti,ab,kf. | 34282 |
| 23 | ((premature or preterm or pre-term) adj3 (birth* or deliver* or labor* or labour*)).ti,ab,kf. | 43466 |
| 24 | ((intrauterine growth adj3 (restrict* or retard*)) or iugr).ti,ab,kf. | 12342 |
| 25 | placenta*.ti,ab,kf. | 98013 |
| 26 | ((pregnancy or gestational) adj3 complication*).ti,ab,kf. | 24746 |
| 27 | (premature rupture adj2 membrane*).ti,ab,kf. | 3666 |
| 28 | exp pregnancy/ and exp toxemia/ | 1169 |
| 29 | prom.ti,ab,kf. | 2392 |
| 30 | (pregnancy adj1 toxemia*).ti,ab,kf. | 3433 |
| 31 | (proteinuria and edema and hypertension and gestosis).ti,ab,kf. | 41 |
| 32 | (EPH adj (toxemia* or gestosis)).ti,ab,kf. | 438 |
| 33 | eclamp*.ti,ab,kf,mp. | 37202 |
| 34 | or/9-33 | 538081 |
| 35 | [and now the summation of microRNA and our outcomes] | 0 |
| 36 | 6 and 34 | 1903 |
| 37 | [human filter] | 0 |
| 38 | animals/ not humans/ | 4516275 |
| 39 | 36 not 38 | 1613 |
| 40 | limit 39 to english | 1533 |

**Ovid Embase <1974 to 2019 March 18>**

| 1 | [jukic microRNA search draft 03 Embase] | 0 |
| --- | --- | --- |
| 2 | [microRNA concept -- if we look at specific ones we'll predetermine the findings, so we're casting a broad net here instead of focusing on 210, 223, 126 etc] | 0 |
| 3 | exp microRNA/ | 127284 |
| 4 | (microRNA* or micro-RNA* or miRNA* or mi-RNA* or miR*).mp. | 240186 |
| 5 | (stRNA* or small temporal rna*).mp. | 53 |
| 6 | or/3-5 | 240219 |
| 7 | [the previous line is the summation of the microRNA concept] | 0 |
| 8 | [and the pregnancy complications of placental origin] | 0 |
| 9 | exp "eclampsia and preeclampsia"/ | 53055 |
| 10 | maternal hypertension/ | 16178 |
| 11 | exp pregnancy complication/ | 112780 |
| 12 | exp "immature and premature labor"/ | 132292 |
| 13 | exp intrauterine growth retardation/ | 39396 |
| 14 | exp low birth weight/ | 56485 |
| 15 | exp Placenta/ | 64382 |
| 16 | (preeclamp* or pre-eclamp*).mp. | 55741 |
| 17 | ((hypertensi* or blood pressure) adj3 (pregnan* or gestation*)).mp. | 20139 |
| 18 | (fetal growth adj (restrict* or retard*)).mp. | 8106 |
| 19 | fgr.mp. | 2621 |
| 20 | (small adj3 gestational age).mp. | 12952 |
| 21 | sga.mp. | 11595 |
| 22 | (low* birthweight* or low* birth weight*).mp. | 57408 |
| 23 | ((premature or preterm or pre-term) adj3 (birth* or deliver* or labor* or labour*)).mp. | 72882 |
| 24 | ((intrauterine growth adj3 (restrict* or retard*)) or iugr).mp. | 33196 |
| 25 | placenta*.mp. | 142858 |
| 26 | ((pregnancy or gestational) adj3 complication*).mp. | 72319 |
| 27 | (premature rupture adj2 membrane*).mp. | 5523 |
| 28 | pregnancy toxemia/ | 2275 |
| 29 | prom.mp. | 4029 |
| 30 | (pregnancy adj1 toxemia*).mp. | 2646 |
| 31 | (proteinuria and edema and hypertension and gestosis).mp. | 66 |
| 32 | (EPH adj (toxemia* or gestosis)).mp. | 527 |
| 33 | eclamp*.mp. | 22933 |
| 34 | or/9-33 | 457697 |
| 35 | [and now the summation of microRNA and our outcomes] | 0 |
| 36 | 6 and 34 | 3268 |
| 37 | [human filter] | 0 |
| 38 | (exp animal/ or exp invertebrate/ or nonhuman/ or animal experiment/ or animal tissue/ or animal model/ or exp plant/ or exp fungus/) not (exp human/ or human tissue/) | 6515144 |
| 39 | 36 not 38 | 2827 |
| 40 | limit 39 to english | 2714 |
| 41 | limit 40 to (conference abstract or conference paper or "conference review") | 852 |
| 42 | 40 not 41 | 1862 |

**Scopus**

| TITLE-ABS-KEY ( microrna* OR micro-rna* OR mirna* OR mi-rna* OR mir* OR strna* OR small-temporal-rna ) | 428376 |
| --- | --- |
| ( TITLE-ABS-KEY ( ( hypertensi* OR blood-pressure ) W/3 ( pregnan* OR gestation* ) ) OR ( fetal-growth W/1 ( restrict* OR retard* ) ) OR ( small W/3 gestational-age ) OR ( low* W/1 birthweight* ) OR ( low* W/1 birth-weight* ) OR ( ( premature OR preterm OR pre-term ) W/3 ( birth* OR deliver* OR labor* OR labour* ) ) OR ( intrauterine-growth W/3 ( restrict* OR retard* ) ) OR ( ( pregnancy OR gestational ) W/3 complication* ) OR ( premature-rupture W/2 membrane* ) OR ( pregnancy W/1 toxemia* ) OR ( eph W/1 ( toxemia* OR gestosis ) ) ) OR ( TITLE-ABS-KEY ( proteinuria AND edema AND hypertension AND gestosis ) ) OR ( TITLE-ABS-KEY ( preeclamp* OR pre-eclamp* OR fgr OR sga OR iugr OR placenta* OR prom OR eclamp* ) ) | 592463 |
| ( TITLE-ABS-KEY ( microrna* OR micro-rna* OR mirna* OR mi-rna* OR mir* OR strna* OR small-temporal-rna ) ) AND ( ( TITLE-ABS-KEY ( ( hypertensi* OR blood-pressure ) W/3 ( pregnan* OR gestation* ) ) OR ( fetal-growth W/1 ( restrict* OR retard* ) ) OR ( small W/3 gestational-age ) OR ( low* W/1 birthweight* ) OR ( low* W/1 birth-weight* ) OR ( ( premature OR preterm OR pre-term ) W/3 ( birth* OR deliver* OR labor* OR labour* ) ) OR ( intrauterine-growth W/3 ( restrict* OR retard* ) ) OR ( ( pregnancy OR gestational ) W/3 complication* ) OR ( premature-rupture W/2 membrane* ) OR ( pregnancy W/1 toxemia* ) OR ( eph W/1 ( toxemia* OR gestosis ) ) ) OR ( TITLE-ABS-KEY ( proteinuria AND edema AND hypertension AND gestosis ) ) OR ( TITLE-ABS-KEY ( preeclamp* OR pre-eclamp* OR fgr OR sga OR iugr OR placenta* OR prom OR eclamp* ) ) ) AND ( LIMIT-TO ( LANGUAGE , "English" ) ) AND ( EXCLUDE ( DOCTYPE , "cp" ) ) AND ( EXCLUDE ( EXACTKEYWORD , "Nonhuman" ) ) | 2259 |

**Updated Search January 2022**

**Medline**

| (microRNA or micro-RNA or miRNA or mi-RNA or miR) AND (Pre-Eclampsia OR preeclampsia OR exp hypertension, pregnancy-induced OR preeclamp OR pre-eclamp OR hypertensi OR blood pressure OR Premature Birth OR Fetal Growth Retardation OR fetal growth restriction OR Low Birth Weight OR small gestational age OR premature birth OR preterm OR pre-term OR placenta) AND ((2020/6/1:2022/1/27[pdat]) AND (english[Filter])) | 413 |
| --- | --- |

**Embase**

| (microrna:ti,ab,kw OR 'micro rna':ti,ab,kw OR mirna:ti,ab,kw OR 'mi rna':ti,ab,kw OR mir:ti,ab,kw) AND ('pre-eclampsia':ti,ab,kw OR preeclampsia:ti,ab,kw OR 'pregnancy induced':ti,ab,kw OR 'blood pressure':ti,ab,kw OR 'gestational hypertension':ti,ab,kw OR 'premature birth':ti,ab,kw OR 'fetal growth retardation':ti,ab,kw OR 'fetal growth restriction':ti,ab,kw OR 'low birth weight':ti,ab,kw OR 'small gestational age':ti,ab,kw OR 'preterm':ti,ab,kw OR 'pre term':ti,ab,kw OR 'placenta':ti,ab,kw) AND [01-06-2020]/sd NOT [27-01-2022]/sd AND [2020-2022]/py | 401 |
| --- | --- |

**Scopus**

| (TITLE-ABS-KEY ( microrna ) OR TITLE-ABS-KEY ( micro-rna ) OR TITLE-ABS-KEY ( mirna ) OR TITLE-ABS-KEY ( mi-rna ) OR TITLE-ABS-KEY ( mir ) ) AND ( TITLE-ABS-KEY ( pre-eclampsia ) OR TITLE-ABS-KEY ( preeclampsia ) OR TITLE-ABS-KEY ( pregnancy-induced ) OR TITLE-ABS-KEY ( blood AND pressure ) OR TITLE-ABS-KEY ( gestational AND hypertension ) OR TITLE-ABS-KEY ( premature AND birth ) OR TITLE-ABS-KEY ( fetal AND growth AND retardation ) OR TITLE-ABS-KEY ( fetal AND growth AND restriction ) OR TITLE-ABS-KEY ( low AND birth AND weight ) OR TITLE-ABS-KEY ( small AND gestational AND age ) OR TITLE-ABS-KEY ( preterm ) OR TITLE-ABS-KEY ( 'pre-term' ) OR TITLE-ABS-KEY ( placenta)) AND ( LIMIT-TO ( PUBYEAR , 2022 ) OR LIMIT-TO ( PUBYEAR , 2021 ) OR LIMIT-TO ( PUBYEAR , 2020 ) ) AND ( LIMIT-TO ( DOCTYPE , "ar" ) ) AND ( LIMIT-TO ( LANGUAGE , "English" ) ) AND ( LIMIT-TO ( SRCTYPE , "j" ) ) | 884 |
| --- | --- |

**Table S2: Characteristics of Included Studies.**

| **Author, Year** | **Study Design** | **Study Site** | **Outcome/Definition** | **Timing of blood draw (weeks)**^1^ | **Method/**  **Sample Type** | **Sample Size** | **miRNA** | **Main Findings** |
| --- | --- | --- | --- | --- | --- | --- | --- | --- |
| Cook et al., 2019  (1) | Prospective study | United Kingdom | Preterm Birth  Defined as spontaneous delivery before 34 weeks gestation. | 12-14 | Genome-Wide  nCounter Assay (Discovery cohort)  RT-qPCR  (Validation cohort)  Plasma | Discovery cohort  Term:16  Preterm:13  Validation cohort  Term:96  Preterm: 14 | hsa-miR-150-5p  hsa-miR-374a-5p  hsa-miR-19b-3p  hsa-miR-185-5p  hsa-miR-15b-5p  hsa-miR-191-5p  hsa-miR-93-5p  hsa-let-7a-5p  hsa-miR-23a-3p | Expression of 9 miRNAs in maternal plasma were significantly different between women preterm and term births. A potential biological biomarker for predicting preterm births could be hsa-miR-150-5p. |
|  |  |  |  |  |  |  |  |  |
| Hromadnikova  et al., 2012  (2) | Retrospective study | Czech Republic | Preeclampsia; Preeclampsia and IUGR; IUGR  Preeclampsia: elevated blood pressure>140/90 mmHg on two measurements taken 4 hours apart or DBP >110 mmHg along with proteinuria >300 mg/24 hours after 20 weeks gestation. Defined using ACOG guidelines.  IUGR: estimated fetal weight below the 10^th^ percentile for the gestational age at evaluation and calculated using the Hadlock formula. Measurements were adjusted according to the population standards in Czech Republic.  Gestational age of outcome: Preeclampsia: ranged from 29-39 weeks^2^.  IUGR: 34 weeks (one case); 33 weeks (one case for preeclampsia and IUGR). | 12 | Targeted  RT-PCR  Plasma | Preeclampsia-16  Preeclampsia and IUGR-5  IUGR-11  Women who later developed preeclampsia and IUGR-7  Healthy Pregnant Controls- 50  Healthy Non-Pregnant Women-10 | miR-516-5p  miR-517*  miR-518b  miR-520a*  miR-520h  miR-525  miR-526a  miR-16  let-7d | No differences in expression of miRNAs in the plasma between healthy pregnant women and those with preeclampsia and IUGR. |
|  |  |  |  |  |  |  |  |  |
| Hromadnikova  et al., 2014  (3) | Prospective cohort study | Czech Republic | Gestational Hypertension  Defined as high blood pressure developing after 20 weeks gestation.  Gestational age of outcome: unknown | 10-13 | Targeted  RT-PCR  Plasma | Gestational Hypertension-18  Healthy Controls- 28 | miR-516-5p  miR-517*  miR-518b  miR-520a*  miR-520h  miR-525 | Four miRNAs measured in the first trimester were significantly higher (miR-516-5p, miR-517*, miR-520h and miR-518b) and associated with risk of gestational hypertension. Measuring only miR-520h or along with miR-518b in the first trimester might help to predict gestational hypertension. |
|  |  |  |  |  |  |  |  |  |
| Hromadnikova  et al., 2017  (4) | Nested case control study | Czech Republic | Preeclampsia; IUGR  Preeclampsia: elevated blood pressure>140/90 mmHg on two measurements taken 4 hours apart along with proteinuria >300 mg/24 hours after 20 weeks gestation. Severe preeclampsia was defined as the onset of one or more of the following features: SBP >160 mmHg or DBP >110 mmHg; proteinuria: >5g of protein in a 24-hour sample; very low urine output (<500 ml in 24 hours); respiratory problems (pulmonary edema or cyanosis); impaired liver function; signs of problems in the central nervous system (severe headache, vision issues); epigastric pain; thrombocytopenia; severe fetal growth restriction. Defined using ACOG guidelines.  IUGR: estimated fetal weight below the 10^th^ percentile for the gestational age at evaluation and calculated using the Hadlock formula. Measurements were adjusted according to the population standards in Czech Republic. IUGR diagnosis also included at least one of the following pathological conditions: abnormal pulsatility index in the umbilical artery, absent or a reversed end diastolic velocity waveform in the umbilical artery, abnormal pulsatility index in the middle cerebral artery, blood flow centralization, amniotic fluid deficiency (anhydramnios and oligohydramnios).  Gestational age of outcome:  Preeclampsia (n=21): <34 weeks (n=6); >34 weeks (n=15)  IUGR (n=18): <34 weeks (n=3); >34 weeks (n=15)). | 10-13 | Targeted  RT-PCR  Plasma | Preeclampsia-21  IUGR-18  Healthy Controls-58 | miR-517-5p  miR-518b  miR-520h  miR-520a-5p  miR-525-5p  miR-516b-5p | Three miRNAs measured in the first trimester were significantly higher (miR-517-5p, miR-518b and miR-520h) and associated with risk of preeclampsia. Measuring miR-517-5p in the first trimester may help to predict preeclampsia. No association between miRNAs and IUGR. |
|  |  |  |  |  |  |  |  |  |
| Jiang et al., 2017  (5) | Retrospective cohort study | China | Severe Preeclampsia  Defined as SBP >140 mmHg or DBP >90 mmHg on two different occasions along with proteinuria (>0.3g/24 hours) after 20 weeks gestation).  Gestational age of outcome:  mean: ~36 weeks | 10-14 | Targeted  RT-PCR  Serum | Severe Preeclampsia-19  Healthy Controls-19 | miR-520g | miR-520g was significantly higher among women with severe preeclampsia than controls. |
|  |  |  |  |  |  |  |  |  |
| Kim et al., 2020 (6) | Prospective cohort study | United Kingdom | IUGR  Defined as birth weight <5^th^ percentile for gestational age according to customized growth charts which were based on parity, gender, ethnicity, and mean height and weight for each ethnic group.  Gestational age of outcome:  Discovery cohort: mean: 37.7 weeks  Validation cohort: mean: 39.2 weeks | 12-14 | Genome-Wide  nCounter Assay (Discovery cohort)  RT-qPCR  (Validation cohort)  Plasma | Discovery Cohort: IUGR: 13 Controls: 16  Validation Cohort: IUGR: 12 Controls: 83 | hsa-miR-374a-5p  hsa-miR-191-5p  hsa-let-7d-5p  hsa-miR-107  hsa-miR-30e-5p hsamiR-4454+miR-7975 | Expression of hsa-miR-374a-5p and hsa-let-7d-5p were significantly higher in women who had IUGR births compared to controls. Combined expression of hsa-miR-374a-5p and hsa-let-7d-5p increased the ability of these miRNAs to predict IUGR. |
|  |  |  |  |  |  |  |  |  |
| Li et al., 2015  (7) | Retrospective cohort study | China | Preeclampsia  SBP >140 mmHg or DBP >90 mmHg on two different occasions along with proteinuria (>0.3 g/24 hours) after 20 weeks gestation).  Gestational age of outcome (n=32):  mean: ~35 weeks  <32 weeks (n=2); >33 weeks (n=30) | 12-14 | Targeted  RT-qPCR  Serum | Preeclampsia-32  Healthy Controls-32 | miR‑152  miR‑182  miR‑183  miR‑210  miR‑1  miR‑328  miR‑363  miR‑377  miR‑500  miR‑584 | No differences in miRNA expression between preeclampsia and controls. |
|  |  |  |  |  |  |  |  |  |
| Li et al., 2020  (8) | Nested case control study | China | Preeclampsia  Defined as new onset of hypertension defined by SBP ≥ 140 mmHg or DBP ≥ 90 mmHg at or after 20 weeks of gestation along with one or more of the following features: proteinuria; maternal organ dysfunction (acute kidney injury, impaired liver function, neurological complications, hematological complications such as thrombocytopenia); uteroplacental dysfunction including fetal growth restriction, abnormal umbilical artery doppler wave form analysis, stillbirth.  Defined using ISSHP guidelines.  Gestational age of outcome (n=32):  mean: ~34 weeks | 12-13 | Genome-Wide  Microarray  Plasma | Preeclampsia-15  Healthy Controls-29 | let-7a-5p  miR-15a-5p  miR-92a-1-3p  miR-106a  miR-125b  miR-130a-3p  miR-191-5p  miR-374a-5p  miR-574-5p  miR-22–5p  miR-93-5p  miR-126-3p  miR-204-3p  miR-365a-3p  miR-559-5p  miR-4264-5p | Sixteen miRNAs were significantly different in preeclampsia compared to controls. Among these miRNAs, expression of 9 miRNAs were higher and 7 miRNAs were lower among preeclamptic women. miR-125b might be used a potential biomarker for predicting preeclampsia |
|  |  |  |  |  |  |  |  |  |
| Li and Liu 2020  (9) | Prospective cohort study | China | IUGR  Defined as fetal birth weight <2500 g and gestational age >37 weeks or actual fetal weight <10^th^ percentile or actual fetal weight below 2 standard deviations of the mean weight compared with sex and gestational age matched fetus.  Gestational age of outcome (n=74):  mean: ~37 weeks | ≤ 13 | Targeted  RT-qPCR  Plasma | IUGR: 74  Non IUGR: 746 | miR-206 | Expression of plasma miR-206 was higher among women who gave birth to a growth restricted infant and might be a potential biomarker to predict IUGR. |
|  |  |  |  |  |  |  |  |  |
| Licini et al., 2021  (10) | Case-Control (Nested in a cohort study) | Italy | Preeclampsia  Defined as blood pressure ≥ 140/90 mmHg on two measurements taken at least 4 hours apart among previously normotensive women along with proteinuria ≥ 300 mg in 24 hours or 2 dip stick readings of at least +2 of midstream or catheter urine specimens if a 24-hour urine collection was not available. Defined using ACOG guidelines.  Gestational age of outcome (n=13):  mean: ~39 weeks | 12 | Targeted  RT-PCR  Plasma | Preeclampsia: 13 Healthy Controls: 18 | miR-125b | Significant association between expression of miR-125b and preeclampsia. Measuring miR-125b in the first trimester might help to predict preeclampsia and act as a potential biomarker. |
|  |  |  |  |  |  |  |  |  |
| Luque et al., 2014  (11) | Nested case-control study | Spain | Preeclampsia  Defined as SBP ≥ 140 mmHg and/or DBP ≥ 90 mmHg on at least two occasions 4 hours apart after 20 weeks gestation among previously normotensive women along with proteinuria >300 mg in a 24-hour urine collection. Defined using ISSHP guidelines.  Gestational age of outcome (n=31): <34 weeks (mean: ~ 31 weeks). | 11 | Genome-Wide  OpenArray  Serum | Preeclampsia-31  Healthy Controls-44 | hsa-miR-126  hsa-miR-127  hsa-miR-221  hsa-miR-942  hsa-miR-143  hsa-miR-125b  hsa-miR-192 | Only serum expression of miR-127 was different between preeclampsia and controls. There were no differences in expression of other miRNAs between the two groups. |
|  |  |  |  |  |  |  |  |  |
| Martinez‑Fierro  et al., 2018  (12) | Nested cohort-case control study | Mexico | Preeclampsia  Severe preeclampsia was defined as SBP ≥ 160 mmHg or DBP ≥ 110 mmHg on two occasions at least six hours apart at bed rest along with proteinuria of 5g in a 24-hour urine collection or ≥ 3+ in two random urine samples collect at least 4 hours apart. Early preeclampsia was defined as onset before 34 weeks gestation and late preeclampsia was an onset after 34 weeks gestation.  Gestational age of outcome (all women with preeclampsia not limited to only those who had a measurement at first trimester): <34 weeks (25% of participants), 34-37 weeks (75% of participants). | 12 | Genome-Wide  TaqMan low density array (TLDA)  Serum | Preeclamspia-6  Healthy Controls-18 | hsa-miR-520d-3p  hsa-miR-518f  hsa-miR-520a  hsa-miR-518b  hsa-miR-520c-3p  hsa-miR-519a  hsa-miR-520f  hsa-miR-521  hsa-miR-523  hsa-miR-518a-3p | There were no differences in expression of miRNA between preeclampsia and controls. |
|  |  |  |  |  |  |  |  |  |
| Martinez‑Fierro  et al., 2019  (13) | Retrospective nested cohort case control study | Mexico | Preeclampsia  Severe preeclampsia was defined as SBP ≥ 160 mmHg or DBP ≥ 110 mmHg on two occasions at least six hours apart at bed rest along with proteinuria of 5g in a 24-hour urine collection or ≥ 3+ in two random urine samples collect at least 4 hours apart. Early preeclampsia was defined as onset before 34 weeks gestation and late preeclampsia was an onset after 34 weeks gestation. Defined using ISSHP guidelines.  Gestational age of outcome (all women with preeclampsia not limited to only those who had a measurement at first trimester): <34 weeks (25% of participants). | 12 | Targeted  qRT-PCR  Serum | Preeclampsia-6  Healthy Controls-18 | hsa-miR-628-3p  hsa-miR-628-5p | Expression of serum hsa-miR-628-3p was significantly higher among women with preeclampsia at 12 weeks than the control group. hsa-miR-628-3p might be a potential biomarker for predicting preeclampsia. |
|  |  |  |  |  |  |  |  |  |
| Martinez‑Fierro  et al., 2021 (14) | Retrospective nested cohort case-control study | Mexico | Preeclampsia  Severe preeclampsia was defined as SBP ≥ 160 mmHg or DBP ≥ 110 mmHg on two occasions at least six hours apart at bed rest along with proteinuria of 5g in a 24-hour urine collection or ≥ 3+ in two random urine samples collect at least 4 hours apart. Early preeclampsia was defined as onset before 34 weeks gestation and late preeclampsia was an onset after 34 weeks gestation. Defined using ISSHP guidelines.  Gestational age of outcome (all women with preeclampsia not limited to only those who had a measurement at first trimester): 34-37 weeks (75% of participants). | 12 | Genome-Wide  TaqMan low density array (TLDA)  Serum | Preeclampsia: 6  Healthy Controls: 18 | hsa-miR-628-3p  hsa-miR-769-5p  hsa-miR-425-5p  hsa-miR-365a-3p  hsa-miR-132-3p  hsa-miR-218-5p | Expression of hsa-miR-628-3p, hsa-miR-769-5p, and hsa-miR-425-5p was significantly different between women with mild preeclampsia and controls; expression of hsa-miR-365a-3p, hsa-miR-132-3p, and hsa-miR-218-5p was different between women with severe preeclampsia and controls.  Measuring expression of hsa-miR-628-3p at 12 weeks might help to predict preeclampsia. |
|  |  |  |  |  |  |  |  |  |
| Mavreli et al., 2020 (15) | Retrospective case-control study | Greece | Preeclampsia  Defined as new onset of hypertension, SBP ≥ 140 mmHg and/or DBP ≥ 90 mmHg on at least two occasions 4 hours to 1 week apart at after 20 weeks of gestation along with proteinuria  ≥ 300 mg in a 24-hour urine collection or two random urine samples collected 4 hours to 1 week apart containing ≥ 1+ by dipstick. Defined using ACOG guidelines. Late onset preeclampsia was diagnosed at or after 34 weeks gestation.  Gestational age of outcome (n=17): median (min, max): 36.4 (34.4-38.5) weeks. | 11-13 | Genome-Wide  Next Generation Sequencing  Plasma | Preeclampsia: 17  Healthy Controls: 17 | miR-99b-5p  miR-23b-5p | Expression of miR-99b-5p and miR-23b-5p was lower in first trimester among women who developed late onset preeclampsia. These miRNAs might act as potential biomarkers for predicting preeclampsia. |
|  |  |  |  |  |  |  |  |  |
| Pei et al., 2021 (16) | Prospective cohort study | China | IUGR  IUGR was defined as fetal birth weight <2500 g and gestational age >37 weeks or actual fetal weight <10^th^ percentile or actual fetal weight below 2 standard deviations of the mean weight compared with sex and gestational age matched fetus.  Gestational age of outcome (n=95):  median (IQR): 37 (36-39) weeks. | 10-11 | Targeted  RT-qPCR  Plasma | IUGR: 95 Controls: 875 | miR-590-3p | No differences in expression of miR-590-3p between IUGR and controls. |
|  |  |  |  |  |  |  |  |  |
| Tang et al., 2021 (17) | Prospective cohort study | China | Preeclampsia  Defined as SBP ≥ 140 mmHg or DBP ≥ 90 mmHg after 20 weeks of gestation among previously normotensive women along with proteinuria ≥ 0.3g in a 24-hour urine collection. Defined using ACOG guidelines.  Gestational age of outcome: unknown | 10-14 | Targeted  qRT-PCR  Serum | Preeclampsia: 30  Healthy Controls: 30 | miR-125b | Expression of miR-125b expressions was significantly higher among preeclamptic women than controls. miR-125b might act as a potential biomarker for predicting preeclampsia. |
|  |  |  |  |  |  |  |  |  |
| Timofeeva et al., 2018  (18) | Retrospective cohort study (includes second cohort) | Russia | Preeclampsia  Defined as blood pressure >140/90 mmHg taken 4 hours apart and proteinuria > 0.3 g/l after 20 weeks gestation. Severe preeclampsia was defined by the onset of the one or more of the following features: SBP >160 mmHg or DBP >110 mmHg; proteinuria >5g/l; very low urine output (<500 ml in 24 hours); respiratory disorders; impaired liver function; epigastric pain; thrombocytopenia; presence of severe growth restriction. Defined using ACOG guidelines.  Gestational age of outcome (n=6): 28-33 weeks (severe early onset preeclampsia). | 11-13 | Targeted^3^  RT-qPCR  Plasma | Preeclampsia-6  Healthy Controls-10 | miR-423-5p  miR-532-5p | Expression of plasma miR-423-5p was higher, and miR-532-5p was lower in the first trimester among women with preeclampsia. |
|  |  |  |  |  |  |  |  |  |
| Ura et al., 2014  (19) | Retrospective study | Italy | Preeclampsia  Definition: not given.  Gestational age of outcome (n=24): median (IQR): 40.6 (39.3-41.1) | 12-14 | Genome-Wide  Microarray  Serum | Preeclampsia-24  Healthy Controls-24 | miR-1233  miR-650  miR-520a  miR-215  miR-210  miR-25  miR-518b  miR-193a-3p  miR-32  miR-204  miR-296-5p  miR-152  miR-126  miR-335  miR-144  miR-668  miR-376a  miR-15b | Expression of 19 miRNAs were significantly associated with preeclampsia. Among these, 12 miRNAs were higher in preeclampsia and 7 miRNAs were lower in preeclampsia. miR-1233 might act as a biomarker for preeclampsia prediction. |
|  |  |  |  |  |  |  |  |  |
| Wang et al., 2021 (20) | Case-Control | China | Preeclampsia  Definition: not given.  Gestational age of outcome: unknown. | 11-12^4^ weeks | Targeted  qRT-PCR  Serum | Preeclampsia: 87  Healthy Controls: 81 | miR-155 | Expression of miR-155 was higher among preeclamptic women than controls. Measurement of miR-155 in the first trimester might act as a potential biomarker for predicting preeclampsia. |
|  |  |  |  |  |  |  |  |  |
| Zhong et al., 2019  (21) | Case-Control | China | Preeclampsia  Defined as blood pressure >140/90 mmHg after 20 weeks of gestation among previously normotensive women along with proteinuria >0.3g in a 24-hour urine collection. Defined using ACOG guidelines.  Gestational age of outcome: unknown. | 11 | Genome-Wide  Microarray  Plasma | Preeclampsia-3  Healthy Controls-3 | hsa‑miR‑1304‑5p  hsa‑miR‑320a  hsa‑miR‑5002‑5p  hsa‑miR‑188‑3p  hsa‑miR‑211‑5p  hiv1‑miR‑TAR‑3p  hsa‑miR‑4498  hsa‑miR‑4432  hsa‑miR‑3184‑5p  hsa‑miR‑92a‑2‑5p  hsa‑miR‑424‑3p  hsa‑miR‑5582‑3p  hsa‑miR‑1273c  hsa‑miR‑3171  hsa‑miR‑203a‑3p  ebv‑miR‑BART1‑5p  hsa‑miR‑5009‑3p  hsa‑miR‑892b  hsa‑miR‑5000‑5p  hsa‑miR‑107  hsa‑miR‑3649  hsa‑miR‑4482‑3p  hsa‑miR‑506‑5p  hsa‑miR‑2392  hsa‑miR‑642b‑3p  hsa‑miR‑4758‑5p  hsa‑miR‑369‑3p  hsa‑miR‑4329  hsa‑miR‑3064-5p | Expression of 29 miRNAs were different between preeclampsia and controls. Among these miRNAs, 3 were higher in preeclampsia, and 26 were lower in preeclampsia. Plasma expression of 3 miRNAs which were higher among women with preeclampsia included hsa‑miR‑1304‑5p, hsa‑miR‑320a, and hsa‑miR‑5002‑5p. |

ACOG, American College of Obstetricians and Gynecologists; DBP, Diastolic Blood Pressure; IQR: Interquartile Range; ISSHP, International Society for the Study of Hypertension in Pregnancy; IUGR, Intrauterine Growth Restriction; SBP, Systolic Blood Pressure.

^1^Represents gestational age; gestational age only for the first trimester is listed for studies which measured miRNAs at multiple timepoints.

^2^Gestational age provided for the 7 women who eventually developed preeclampsia.

^3^Only includes the miRNA examined in the second cohort of women where blood was drawn in the first trimester.

^4^Not clearly specified; Median gestational age at enrollment was between 11-12 weeks.

**References**

1. Cook, J., P.R. Bennett, S.H. Kim, et al., First Trimester Circulating MicroRNA Biomarkers Predictive of Subsequent Preterm Delivery and Cervical Shortening. Scientific Reports, 2019. 9(1): p. 5861.

2. Hromadnikova, I., K. Kotlabova, J. Doucha, K. Dlouha, and L. Krofta, Absolute and relative quantification of placenta-specific micrornas in maternal circulation with placental insufficiency-related complications. Journal of Molecular Diagnostics, 2012. 14(2): p. 160-7.

3. Hromadnikova, I., K. Kotlabova, L. Hympanova, J. Doucha, and L. Krofta, First trimester screening of circulating C19MC microRNAs can predict subsequent onset of gestational hypertension. PLoS ONE [Electronic Resource], 2014. 9(12): p. e113735.

4. Hromadnikova, I., K. Kotlabova, K. Ivankova, and L. Krofta, First trimester screening of circulating C19MC microRNAs and the evaluation of their potential to predict the onset of preeclampsia and IUGR. PLoS ONE [Electronic Resource], 2017. 12(2): p. e0171756.

5. Jiang, L., A. Long, L. Tan, et al., Elevated microRNA-520g in pre-eclampsia inhibits migration and invasion of trophoblasts. Placenta, 2017. 51: p. 70-75.

6. Kim, S.H., D.A. MacIntyre, R. Binkhamis, et al., Maternal plasma miRNAs as potential biomarkers for detecting risk of small-for-gestational-age births. EBioMedicine, 2020. 62: p. 103145.

7. Li, Q., A. Long, L. Jiang, et al., Quantification of preeclampsia-related microRNAs in maternal serum. Biomedical Reports, 2015. 3(6): p. 792-796.

8. Li, Q., Y. Han, P. Xu, et al., Elevated microRNA-125b inhibits cytotrophoblast invasion and impairs endothelial cell function in preeclampsia. Cell death discovery, 2020. 6: p. 35.

9. Li, Y. and J. Liu, MicroRNA-206 predicts raised fetal growth retardation risk through the interaction with vascular endothelial growth factor in pregnancies. Medicine, 2020. 99(7): p. e18897.

10. Licini, C., C. Avellini, E. Picchiassi, et al., Pre-eclampsia predictive ability of maternal miR-125b: a clinical and experimental study. Transl Res, 2021. 228: p. 13-27.

11. Luque, A., A. Farwati, F. Crovetto, et al., Usefulness of circulating microRNAs for the prediction of early preeclampsia at first-trimester of pregnancy. Scientific Reports, 2014. 4: p. 4882.

12. Martinez-Fierro, M.L., I. Garza-Veloz, C. Gutierrez-Arteaga, et al., Circulating levels of specific members of chromosome 19 microRNA cluster are associated with preeclampsia development. Archives of Gynecology & Obstetrics, 2018. 297(2): p. 365-371.

13. Martinez-Fierro, M.L., J.G. Carrillo-Arriaga, M. Luevano, et al., Serum levels of miR-628-3p and miR-628-5p during the early pregnancy are increased in women who subsequently develop preeclampsia. Pregnancy Hypertension, 2019. 16: p. 120-125.

14. Martinez-Fierro, M.L. and I. Garza-Veloz, Analysis of Circulating microRNA Signatures and Preeclampsia Development. Cells, 2021. 10(5).

15. Mavreli, D., A. Lykoudi, G. Lambrou, et al., Deep Sequencing Identified Dysregulated Circulating MicroRNAs in Late Onset Preeclampsia. In Vivo, 2020. 34(5): p. 2317-2324.

16. Pei, J., Y. Li, Z. Min, et al., MiR-590-3p and its targets VEGF, PIGF, and MMP9 in early, middle, and late pregnancy: their longitudinal changes and correlations with risk of fetal growth restriction. Ir J Med Sci, 2021.

17. Tang, J., D. Wang, J. Lu, and X. Zhou, MiR-125b participates in the occurrence of preeclampsia by regulating the migration and invasion of extravillous trophoblastic cells through STAT3 signaling pathway. J Recept Signal Transduct Res, 2021. 41(2): p. 202-208.

18. Timofeeva, A.V., V.A. Gusar, N.E. Kan, et al., Identification of potential early biomarkers of preeclampsia. Placenta, 2018. 61: p. 61-71.

19. Ura, B., G. Feriotto, L. Monasta, et al., Potential role of circulating microRNAs as early markers of preeclampsia. Taiwanese Journal of Obstetrics & Gynecology, 2014. 53(2): p. 232-4.

20. Wang, Z., Y. Shan, Y. Yang, T. Wang, and Z. Guo, MicroRNA-155 is upregulated in the placentas of patients with preeclampsia and affects trophoblast apoptosis by targeting SHH/GLi1/BCL2. Hum Exp Toxicol, 2021. 40(3): p. 439-451.

21. Zhong, Y., F. Zhu, and Y. Ding, Differential microRNA expression profile in the plasma of preeclampsia and normal pregnancies. Experimental and Therapeutic Medicine, 2019. 18(1): p. 826-832.


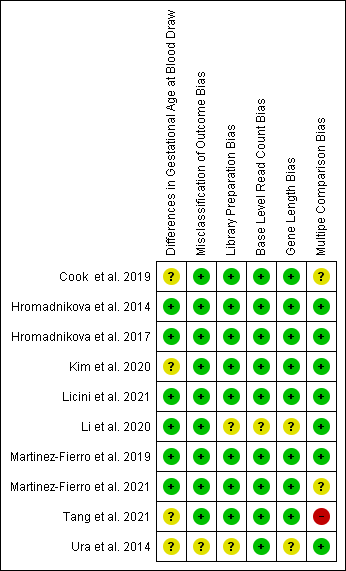


**Figure S1. Risk of bias in the studies which had the most replications.**

**+, Low risk of bias; -, High risk of bias; ?, Unclear risk of bias.**
