# Supplementary figures and images for: Circulating miRNAs in the first trimester and pregnancy complications: a systematic review
Source: Epigenetics. 2022 Dec 12;18(1):2152615. doi: 10.1080/15592294.2022.2152615 (PMC9980650; doi:10.1080/15592294.2022.2152615)

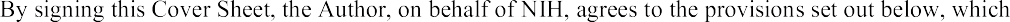

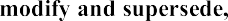

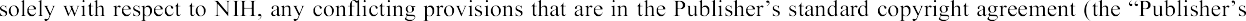

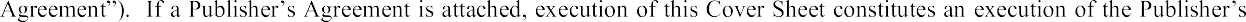

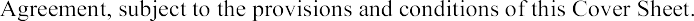

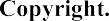

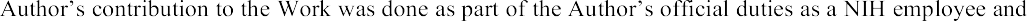

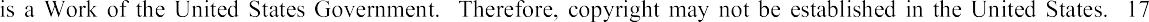

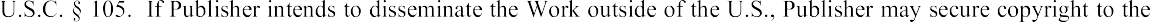

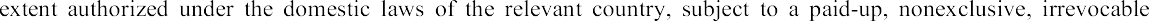

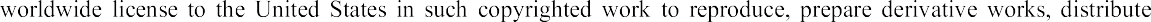

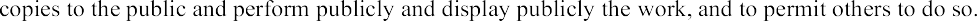

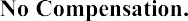

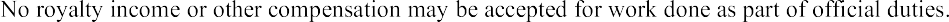

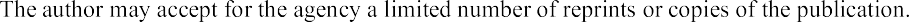

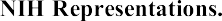

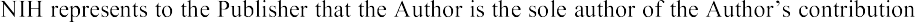

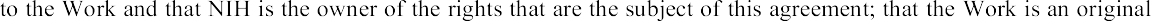

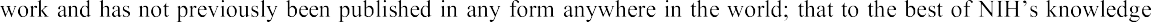

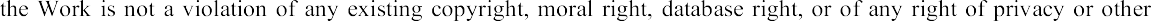

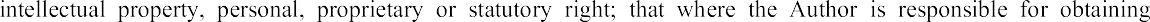

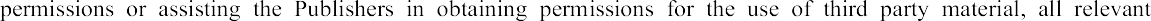

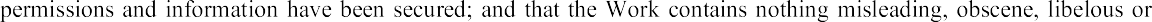

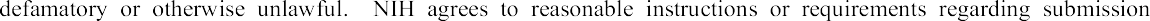

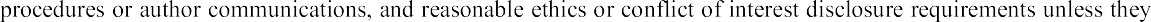

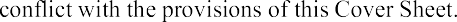

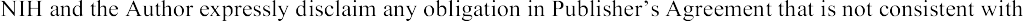

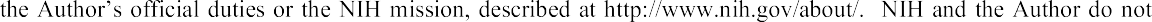

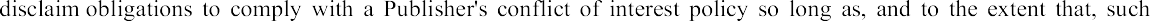

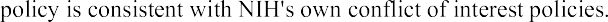

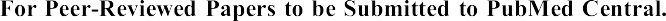

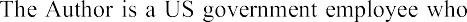

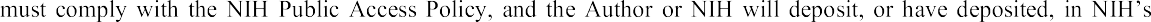

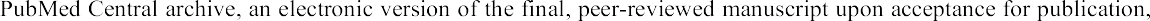

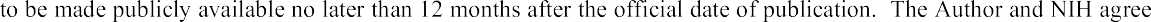

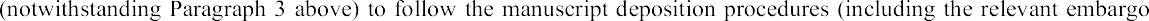

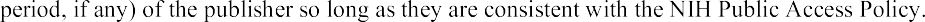

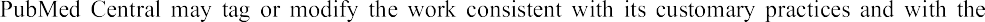

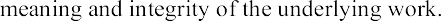

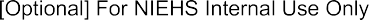

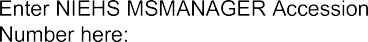


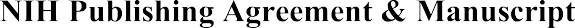

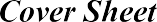


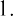

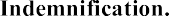

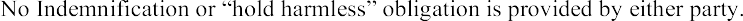

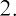

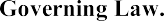

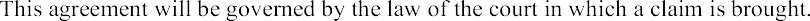

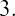


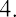


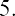


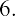

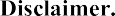


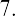


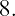

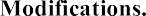


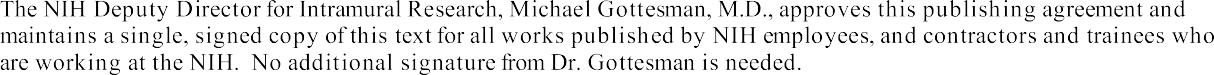

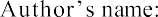

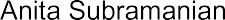

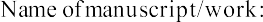

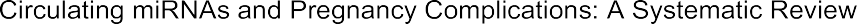

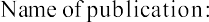

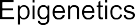

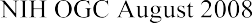


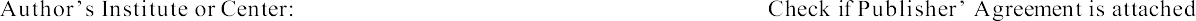

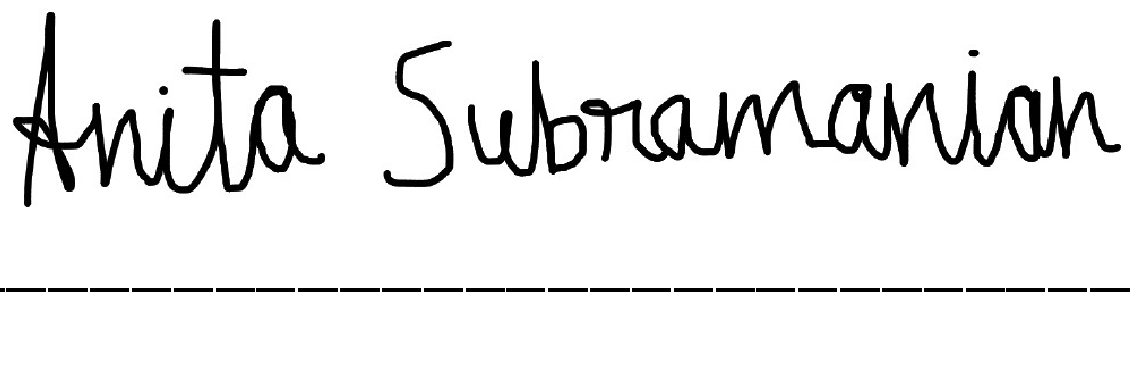

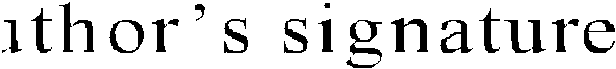

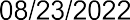

Supplement: Supplemental Material [file KEPI_A_2152615_SM2135.zip › supplement/NIH Cover Sheet.docx]
